# Supplementary material for: Motor phenotypes of amyotrophic lateral sclerosis – a three-determinant anatomical classification based on the region of onset, propagation of motor symptoms, and the degree of upper and lower motor neuron dysfunction
Source: Neurol Res Pract. 2025 Apr 28;7(1):27. doi: 10.1186/s42466-025-00389-w (PMC12036282; doi:10.1186/s42466-025-00389-w)
Supplement: Supplementary file 1 — Supplementary Material 1 [file 42466_2025_389_MOESM1_ESM.pdf]

OPM classification of ALS motor phenotypes – commentary for the use in clinical practice and research

Version 3.1

Short name	Code	Commentary
**Onset**	**O**	**Onset region**
head onset	O1	Onset with dysarthria or dysphagia, also named “bulbar onset”
distal arm onset	O2d	Onset with weakness or slowed, poorly coordinated voluntary movements at the distal arm (hand)
proximal arm onset	O2p	Onset with weakness at proximal arm (shoulder)
trunk respiratory onset	O3r	Onset with weakness at the trunk with hypoventilation
trunk axial onset	O3a	Onset with weakness of trunk muscles without concurrent hypoventilation
distal leg onset	O4d	Onset with weakness or slowed, poorly coordinated voluntary movements at the distal leg (foot)
proximal leg onset	O4p	Onset with weakness or slowed, poorly coordinated voluntary movements at the proximal leg (hip and thigh muscles)
**Propagation**	**P**	**Propagation (“spreading”)**
Earlier propagation	PE	Propagation of slowed, poorly coordinated voluntary movements or weakness from the region of onset to another vertically distant body region *within 12 months* of symptom onset.
Later propagation	PL	Propagation of slowed, poorly coordinated voluntary movements or weakness from the region of onset to another, vertically distant body region *later than 12 months* of symptom onset. It includes O1, PL (also named "progressive bulbar paralysis, PBP)", O2d/p, PL (also named "flail arm-syndrome"), and O4d/p, PL (also named "flail leg-syndrome.”
**Propagation not classifiable**	PN	Propagation of slowed, poorly coordinated voluntary movements or weakness from the region of onset to another, vertically distant body region not yet classifiable as time since symptom onset is less than 12 months.
**Motor neuron dysfunction**	**M**	**Degree of upper and/or lower motor neuron dysfunction**
balanced motor neuron dysfunction	M0	Balanced combined symptoms of the upper motor neuron (slowed, poorly coordinated voluntary movements, increased reflexes and/or spasticity) and the lower motor neuron (weakness and associated atrophy), also named “classic ALS”
dominant upper motor neuron (UMN) dysfunction	M1d	Dominant symptoms of the upper motor neuron (slowed, poorly coordinated voluntary movements, increased reflexes and/or spasticity) and only discrete symptoms of the lower motor neuron (weakness and associated atrophy)
pure UMN dysfunction	M1p	Pure symptoms of the upper motor neuron (slowed, poorly coordinated voluntary movements, increased reflexes and/or spasticity) and no evidence of lower motor neuron symptoms (weakness and associated atrophy); also named “primary lateral sclerosis (PLS)” after a disease duration of 48 months

dominant lower motor neuron (LMN) dysfunction	M2d	Dominant symptoms of the lower motor neuron (weakness and associated atrophy) and only discrete symptoms of the upper motor neuron (slowed, poorly coordinated voluntary movements, increased reflexes and/or spasticity)
pure LMN dysfunction	M2p	Pure symptoms of the lower motor neuron (weakness and associated atrophy) and only discrete symptoms of the upper motor neuron (slowed, poorly coordinated voluntary movements, increased reflexes and/or spasticity); also named “progressive muscle atrophy (PMA)” after a disease duration of 48 months
dissociated motor neuron dysfunction	M3	Dominant symptoms of the lower motor neuron (weakness and associated atrophy) in the arms and dominant symptoms of the upper motor neuron (slowed, poorly coordinated voluntary movements, increased reflexes and/or spasticity) in the legs
