# Supplementary material for: Motor phenotypes of amyotrophic lateral sclerosis – a three-determinant anatomical classification based on the region of onset, propagation of motor symptoms, and the degree of upper and lower motor neuron dysfunction
Source: Neurol Res Pract. 2025 Apr 28;7(1):27. doi: 10.1186/s42466-025-00389-w (PMC12036282; doi:10.1186/s42466-025-00389-w)
Supplement: Supplementary file 2 — Supplementary Material 2 [file 42466_2025_389_MOESM2_ESM.pdf]

OPM-Klassifikation der motorischen ALS-Phänotypen – Kommentierung für die klinische Anwendung und Forschung

Version 3.1

Kurzname	Code	Erklärung
**Symptombeginn**	**O**	**Region des Symptombeginns**
Beginn am Kopf	O1	Beginn mit Sprech- oder Schluckstörung; auch „bulbärer Beginn“ genannt
Beginn am distalen Arm	O2d	Beginn mit Schwäche oder verlangsamten und unkoordinierten Bewegungen am distalen Arm (Hand)
Beginn am proximalen Arm	O2p	Beginn mit Schwäche am proximalen Arm (Schulter)
Beginn am Rumpf mit Atemschwäche	O3r	Beginn am Rumpf mit Atemschwäche
Beginn mit Rumpfinstabilität	O3a	Beginn mit Schwäche der Rumpfmuskulatur ohne gleichzeitige Atemschwäche
Beginn am distalen Bein	O4d	Beginn mit Schwäche oder verlangsamten und unkoordinierten Bewegungen am distalen Bein (Fuß)
Beginn am proximalen Bein	O4p	Beginn mit Schwäche oder verlangsamten und unkoordinierten Bewegungen am proximalen Bein (Hüft- und Oberschenkelmuskulatur)
**Propagation**	**P**	**Propagation (“Ausbreitung”)**
Frühe **Propagation**	PE	Ausbreitung von Schwäche oder verlangsamten und unkoordinierten Bewegungen auf andere, vertikale Regionen innerhalb von 12 Monaten
Späte Propagation	PL	Ausbreitung von Schwäche oder verlangsamten und unkoordinierten Bewegungen auf andere, vertikale Regionen nach 12 Monaten oder später; umfasst O1, PL (auch Progressive Bulbärparalyse genannt), O2d/p, PL (auch „Flail-Arm-Syndrom“ genannt), and O4d/p, PL (auch „Flail-Leg-Syndrom“ genannt).
Noch nicht klassifizierbare Propagation	PN	Ausbreitung von Schwäche oder verlangsamten und unkoordinierten Bewegungen auf andere, vertikale Regionen noch nicht erkennbar, aber seit Symptombeginn noch keine 12 Monate vergangen
**Motoneuron-Symptome**	**M**	**Symptome des ersten und/oder zweiten Motoneurons**
Kombinierte Symptome des ersten und zweiten Motoneurons	M0	Ausbalancierte, kombinierte Symptome des ersten Motoneurons (verlangsamte und unkoordinierte Bewegungen, gesteigerte Reflexe sowie Spastizität) und des zweiten Motoneurons (Muskelschwäche und Muskelschwund), auch „klassische ALS“ genannt
Dominante Symptome des ersten Motoneurons	M1d	Dominante Symptome des ersten Motoneurons (verlangsamte und unkoordinierte Bewegungen, gesteigerte Reflexe und/oder Spastizität) und nur diskrete Symptome des zweiten Motoneurons (Muskelschwäche und Muskelschwund)
Ausschließliche („pure“) Symptome	M1p	Ausschließliche („pure“) Symptome des ersten Motoneurons (verlangsamte und unkoordinierte Bewegungen, gesteigerte Reflexe und/oder Spastizität) und keine Symptome des zweiten

des ersten Motoneurons		Motoneurons (Muskelschwäche und Muskelschwund); nach Krankheitsverlauf von 48 Monaten auch „Primäre Lateralsklerose (PLS)“ genannt
Dominante Symptome des zweiten Motoneurons	M2d	Dominante Symptome des zweiten Motoneurons (Muskelschwäche und Muskelschwund) und nur diskrete Symptome des ersten Motoneurons (verlangsamte und unkoordinierte Bewegungen, gesteigerte Reflexe und/oder Spastizität)
Ausschließliche („pure“) Symptome des zweiten Motoneurons	M2p	Ausschließliche („pure“) Symptome des zweiten Motoneurons (Muskelschwäche und Muskelschwund) und nur diskrete Symptome des ersten Motoneurons (verlangsamte und unkoordinierte Bewegungen, gesteigerte Reflexe und/oder Spastizität); nach Krankheitsverlauf von 48 Monaten „Progressive Muskelatrophie (PMA)“ genannt
Dissoziierte Symptome des ersten und zweiten Motoneurons	M3	Dominante Symptome des zweiten Motoneurons (Muskelschwäche und Muskelschwund) an den Armen und dominante Symptome des ersten Motoneurons (verlangsamte und unkoordinierte Bewegungen, gesteigerte Reflexe und/oder Spastizität) an den Beinen; auch „Brachial-atroph paraspastischer Phänotyp genannt
